# The Use of Cone Beam Computed Tomography (CBCT) to Determine Supernumerary and Impacted Teeth Position in Pediatric Patients: A Case Report

**DOI:** 10.5681/joddd.2013.008

**Published:** 2013-02-21

**Authors:** Hossein Nematolahi, Hamed Abadi, Zahra Mohammadzade, Mostafa Soofiani Ghadim

**Affiliations:** ^1^Associate Professor, Department of Pediatric Dentistry, Mashhad University of Medical Sciences, Mashhad, Iran; ^2^Postgraduate Student, Department of Pediatric Dentistry, Mashhad University of Medical Sciences, Mashhad, Iran

**Keywords:** CBCT, impacted teeth, odontoma, pediatric dentistry, supernumerary teeth

## Abstract

A case of a compound odontoma which caused delayed eruption of right maxillary central incisor in a ten year old girl is presented with clinical and radiographic findings. The patient presented with complaint of a hard painless swelling in the right anterior region of the maxilla and absence of right maxillary central incisor. After clinical examination, periapical and occlusal radiographs of the mentioned region were taken. Impacted maxillary right central incisor was seen malformed shape on the intraoral radiographs. After taking a cone beam computed tomography (CBCT) a compound odontoma associated with the labial aspect of impacted maxillary right central incisor was diagnosed and then removed by simple local excision under local anesthesia. The removal of the odontoma was followed by forced eruption of the impacted central incisor. After three months the tooth returned to its original position.

## Introduction

The periapical and occlusal radiographs are among intraoral radiography techniques which have long been used for diagnostic purposes. Due to superimposing structural components, the correct diagnosis of the location of a lesion is sometimes impossible with these techniques. It is now possible to acquire three-dimensional (3D) images of the oral and maxillofacial structures by cone beam computed tomography (CBCT) on a high resolution of 0.001 mm^3^ voxels, and these 3D images can provide a better understanding of many anatomical structures, as well as pathologic conditions, developmental anomalies, and traumatic injuries.^1^ CBCT utilizes an extraoral imaging scanner, which was developed in the late 1990’s to produce three-dimensional scans of the maxillofacial skeleton at a considerably lower radiation dose than conventional computed tomography (CT).^2^ CBCT is based on a cone-shaped X-ray beam centered on a two-dimensional detector.^3^ This technique can be used in pediatric dentistry for the study of special cases such as determining the supernumerary and impacted teeth position.^4^ The advantages of CBCT include 3D imaging of dental structures, less imaging time in comparison to computerized tomography (CT), easy data transfer, and less scattered radiation.^4-5^

Supernumerary tooth is a tooth anomaly that may cause failure of adjacent teeth to erupt, displacement and crowding of the adjacent teeth, abnormal diastema, root resorption, and even dentigerous cyst formation. CBCT offers an undistorted view of the dentition that shows the details of individual dental morphology, including missing, supernumerary and anomalous teeth, as well as the 3-D spatial orientation of the teeth and roots. CBCT imaging enables the clinician to evaluate eruption patterns and their variations with an improved accuracy compared to that of conventional radiographs.^6^

The use of CBCT has proven useful in the management of children with supernumerary teeth. CBCT images can be used to locate the precise position of impacted and supernumerary teeth and to make an accurate diagnosis and design treatment strategies that would result in less invasive surgical intervention. The present paper reports one such case considering the advantages of CBCT scan in exact diagnosis and the presentation of effective treatment plans for pediatric dentists.

## Case report

A 10-year old girl with uneruption of maxillary right central incisor was referred to the Department of Pediatric Dentistry, Mashhad University of Medical Sciences, Iran. There was no considerable point in the medical records, but a traumatic bicycle accident at the age of three affecting anterior maxilla. A surgical incision in the gum tissue had been performed by a general dentist to accelerate the eruption of the unerupted tooth six months before. In clinical examination, a swelling was evident in the right anterior maxilla with a dimension of 1 cm × 1 cm, hard in consistency and in gingiva color, with no sign of spontaneous pain or by palpation (Figure 1A). Adjacent teeth had no sign of decay or pain during the percussion and there was enough space for the eruption of the impacted teeth. In the periapical radiographic view, the crown of the impacted central incisor appeared to be malformed, which could be explained by the history of trauma. The lack of spontaneous eruption could also be justified; however, the possibility of existence of a hard calcified mass separate from the tooth was suspected due to a likely radiographic superimposition on the crown of the unerupted tooth. For an accurate diagnosis, occlusal radiograph was requested with a 60-degree angle of radiation (Figure 2A) with the resulting image showing no separation between the opaque mass and the malformed crown. Following the failure to achieve an accurate diagnosis, CBCT scans were taken on the Planmeca ProMax 3D (Planmeca, Helsinki, Finland) which revealed a closely-situated hard calcified tissue mass separate from the unerputed tooth, with exact dimensional details. In sagittal plan, the mass was seen to be covered only by soft tissue, and the healthy impacted incisor crown with normal anatomy could be observed (Figure 3A).


Figure 1. Clinical appearance of lesions, swelling in the anterior maxilla (A). Unerupted teeth in occlusal viewradiograph (B). Unerupted tooth and odontoma in CBCT scan (sagittal view, C). Beginning of orthodontic forced eruption treatment (D). End of forced eruption treatment (E).

**a:**
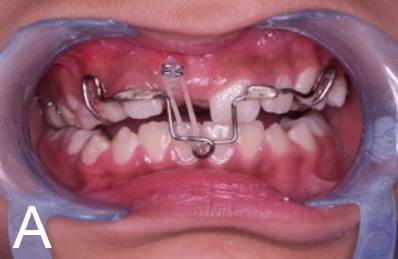

**b:**
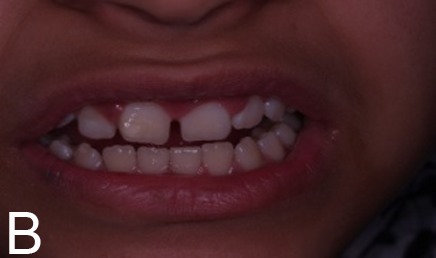

**c:**
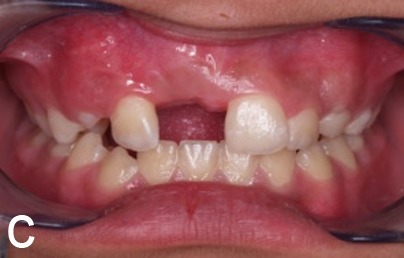

**d:**
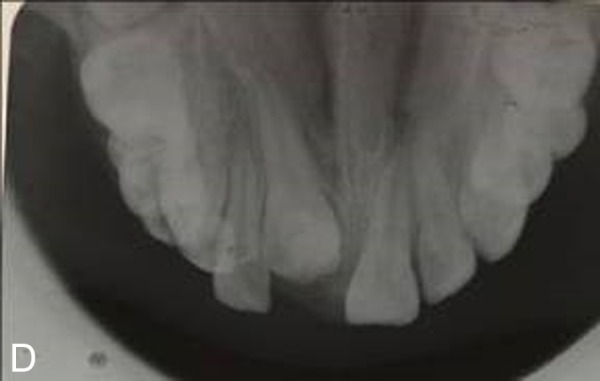

**e:**
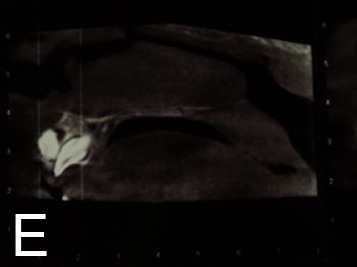

Because of the closed apex of the impacted tooth and lack of spontaneous eruption, forced eruption was considered as treatment. The treatment plan included a surgrical incision with a trap door window approach and cutting out the calcified tissue mass, which was found to be a compound odontoma in pathology report. One week after removal of the tissue mass, a working model was prepared and an acrylic appliance was designed including retentive components and labial bow, the loop of which was located about 2 mm below the incisal edge of tooth #11. After preparing the acrylic appliance, a suitable bracket was attached to the buccal surface of the exposed crown of the impacted central incisor (Figure 4A) and Heavy 3/16 Smile Safari elastics (Ortho Organizer, Germany) were used to put force onto the teeth. The patient was advised for every 3-day replacement of the elastic. After three months, the tooth had moved to its original position (Figure 5A).

## Discussion

The present case highlights the superiority of CBCT images compared with conventional intraoral radiographs with regards to surgical treatment planning of impacted and supernumerary teeth. In the present case, the findings of periapical and occlusal radiographic images in the maxillary anterior region revealed the existence of an impacted maxillary right central incisor with malformed crown. CBCT, however, showed the existence of an odontoma in close proximity to the crown of the impacted teeth, which possessed normal anatomy.

Supernumerary teeth may have a normal morphology or may be rudimentary and miniature. Supernumeraries might occur singly or multiply and in any region of the jaws. They are most commonly conical in shape and most of them occur in the premaxillary region.^7^ Supernumerary teeth are classified according to their form and location. There is a greater variety of forms presenting in the permanent dentition. Four different morphological types of supernumerary teeth have been described. Howard lists odontoma as the fourth category of supernumerary tooth.^8^ Odontomas are the most common of the odontogenic tumors of the jaws which are benign, slow growing and non-aggressive. Odontomas are usually asymptomatic but sometimes may interfere with the eruption of the associated tooth leading to impaction or delayed eruption. These lesions are usually diagnosed on routine radiological examination in the second decade of the life.^9^

Detection of supernumeraries is best achieved by thorough clinical and radiographic examination. It seems that for the treatment planning of the supernumerary and impacted teeth, the exact diagnosis of anatomical shape and position of supernumeraries have great importance. Radiographs are helpful in evaluating the position and nature of these anomalies. Traditional two-dimensional radiographs include a panoramic radiograph to evaluate the vertical position, an occlusal x-ray to evaluate the proximity to adjacent teeth, and periapical radiographs to determine the labiopalatal position, but volumetric images of the maxillary dentition are obtained from a CBCT scan.^10^

In our case, the crown of the impacted right maxillary central incisor was seen malformed in periapical and occlusal radiographs, a fact that which seemed in accordance with a history of trauma to the region. However, neither of the two dimensional radiographic techniques were able to accurately depict the size or anatomy of the tooth. We requested a CBCT scan to achieve a more detailed assessment of the shape and position of the impacted tooth. The scan revealed a calcified mass separate from the impacted tooth in close proximity, and provided exact dimensional details.

The use of diagnostic help resulting from the application of conventional radiological techniques with CBCT has been studied in order to determine the position of supernumerary and impacted teeth.^4^ The results show that both techniques are useful as initial diagnostic tools but more detailed information about the position of the teeth, the presence or absence of root resorption and treatment planning are provided with CBCT images.^4^ The finding of Liu et al^7^ also confirm these findings. The author of the latter study recommend that CBCT be used routinely for the treatment of supernumerary teeth, especially for those cases with multiple supernumeraries, those with local malocclusions, or with high-situated supernumeraries,^7^ but Tumen et al^11^ emphasize that before requesting a CBCT, the necessities of this scan and risk/advantage analysis need to be determined.

Although at present the availability of the CBCT technique as a useful diagnostic tool for dentists cannot be neglected, this method may not be a good choice for initial evaluations. On the other hand, CBCT has its specific limitations; to perform this technique, the patient must remain motionless during the scan, and the financial cost of this imaging technique can also be a concern. In addition, relatively high amount of radiation received by the patients in this scan is equivalent to a full-mouth intra-oral radiographic examination. However, the radiation dose to the patient with CBCT is markedly lower than that of multi-slice CT; doses are 3 to 7 times more than panoramic doses and 40% less than conventional CT.^12-13^ Considering only the radiation dose, the use of a CBCT image is not recommended routinely in pediatric dentistry. Therefore, the decision making for radiographic examination is a balance between the risk assessment and the diagnostic information needed. When additional information is necessary, which is the case in patients with impacted teeth, dental resorption, ankylosis, temporomandibular joint evaluation, or surgical planning, CBCT should be the method of choice.^13^

It seems that for treatment planning of supernumerary and impacted teeth, the exact diagnosis of anatomical shape and position of the mentioned teeth is an important issue. Two different radiographic diagnostic methods are selected. First, conventional radiography is performed with intra- and extra-oral radiographs, including a panoramic, upper occlusal or periapical radiography. If definitive diagnosis is not obtained, CBCT scans are recommended in the second stage.

## Conclusion

In pediatric dentistry, the application of CBCT technique can be helpful in detecting the exact location of supernumerary and impacted teeth and in appropriate treatment planning. However, advantages and disadvantages of CBCT must be considered together, and only when more information is need, the use of this technique is suggested. Its unnecessary prescription should otherwise be avoided.
